# Evaluation of Torquetenovirus (TTV) Particle Integrity Utilizing PMAxx™

**DOI:** 10.3390/ijms26136542

**Published:** 2025-07-07

**Authors:** Giuseppe Sberna, Claudia Minosse, Cosmina Mija, Eliana Specchiarello, Pietro Giorgio Spezia, Sara Belladonna, Giulia Berno, Lavinia Fabeni, Giulia Matusali, Silvia Meschi, Daniele Focosi, Fabrizio Maggi

**Affiliations:** 1 Laboratory of Virology and Biosafety Laboratories, National Institute for Infectious Diseases Lazzaro Spallanzani-IRCCS, 00149 Rome, Italy; 2 North-Western Tuscany Blood Bank, Pisa University Hospital, 56100 Pisa, Italy

**Keywords:** Torquetenovirus, genome quantification, COVID-19 vaccination, propidium monoazide (PMA)

## Abstract

Torquetenovirus (TTV) is a ubiquitous, non-pathogenic DNA virus that has been suggested as a biomarker of immune competence, with the viral load correlating with the level of immunosuppression. However, by detecting non-intact viral particles, standard PCR-based quantification may overestimate the TTV viremia. To improve the clinical relevance of TTV quantification, in this study, we investigated the use of PMAxx™, a virion viability dye that selectively blocks the amplification of compromised virions. Serum samples from 10 Hepatitis C Virus-positive (HCV+) individuals, 81 liver transplant recipients (LTRs), and 40 people with HIV (PWH) were treated with PMAxx™ and analyzed for TTV DNA loads by digital droplet PCR (ddPCR). Furthermore, anti-SARS-CoV-2 IgG levels and neutralizing antibody (nAbs) titers were measured post-COVID-19 vaccination. Using ddPCR, the PMAxx™ treatment significantly reduced the TTV DNA levels in all the groups (mean reduction: 0.66 Log copies/mL), indicating the abundant presence of non-intact, circulating viral genomes. However, correlations between TTV DNA and SARS-CoV-2 IgG or nAbs were weak or absent in both PMAxx™-treated and untreated samples. These findings suggest that while PMAxx™ enhanced the specificity of TTV quantification, it did not improve the predictive value of TTV viremia at assessing vaccine-induced humoral responses.

## 1. Introduction

Torquetenovirus (TTV) is a common, unenveloped, circular, single-stranded DNA virus belonging to the Anelloviridae family [1] and characterized by a very high prevalence worldwide [2], in both humans and animals [3,4,5], with notable differences in viral species and genotypes observed across different hosts [2,3,4,5]. Although considered non-pathogenic in immunocompetent individuals [6], the kinetics of TTV replication are closely linked to the host immune function, making it a potential biomarker for immunosuppression [7]. Recent findings show that TTV DNA measurement is useful for monitoring immune responses in immunocompromised individuals, such as organ transplant recipients and people with HIV (PWH) [8,9,10], for evaluating immune reconstitution after transplantation and chemotherapy [11], and for predicting humoral and cell-mediated immune responses to SARS-CoV-2 mRNA vaccines [7,8]. Notably, a healthy control group was included in a previous study [4], where only the total TTV DNA was measured. The results show no significant correlation between the TTV load and humoral immune responses to SARS-CoV-2 vaccination, suggesting that TTV may not serve as a useful biomarker in an immunocompetent population [8]. Furthermore, TTV is becoming a promising marker for tracking immunosuppressive therapy in recipients of solid organ transplants, where viral reactivation indicates the degree of immune suppression [11,12]. TTV has also been investigated in relation to sepsis [13], autoimmune diseases [14], and cancer immunotherapy [15], which support its potential as a universal immune marker [16].

TTV DNA is detected by traditional molecular techniques, such as quantitative PCR (qPCR) and digital droplet PCR (ddPCR) [9], which work irrespective of the TTV structural integrity, thus potentially resulting in an overestimation of the circulating viral DNA. Thus, to more precisely determine the immune system status and immunosuppressive treatments’ effectiveness, an accurate measurement of TTV load is crucial, and methodologies able to specifically quantify the viral DNA included in complete particles only are needed.

To address this point, several methods can be employed to identify intact viral particles, including size-exclusion chromatography [17], anion-exchange chromatography [18], and viability dyes [19]. Among the latter, propidium monoazide (PMA) and its enhanced derivative PMAxx™ selectively penetrate compromised viral capsids, thus stopping the amplification of non-intact viral genomes during PCR assays [19,20,21], offering a faster, simpler, and more accessible alternative workflow. Notably, treatment with a concentration of 50 μM PMAxx™, combined with ddPCR, has been shown to discriminate between intact and non-intact viruses [19,20], and to reveal how freezing may affect the viral integrity [20].

These dyes have been applied in bacteriophage and viral integrity studies, including research on enteric viruses [22,23], respiratory viruses [19], and Monkeypox virus [20]. Nonetheless, the potential of PMAxx™ in TTV assessment remains unexplored, prompting further research to determine whether it could enhance the precision of TTV DNA measurements.

If PMAxx™-based assays enhance the specificity of viral DNA detection, they could help prevent misinterpretations in immune competence evaluations and thereby improve clinical decision-making. For this reason, a TTV–PMAxx™ assay was developed and ap-plied in this study, and the correlation between the TTV levels (both with and without the PMAxx™ treatment) and the SARS-CoV-2 anti-Spike RBD IgG or neutralizing antibody (nAbs) responses was assessed.

## 2. Results

### 2.1. TTV DNA in Fresh and Frozen Samples with or Without PMAxx™ Treatment

For the method development, PMAxx™ was employed to assess the integrity of TTV in both fresh samples (10 samples from Hepatitis C Virus positive (HCV+) patients) and frozen samples (81 and 40 samples from the liver transplant recipient (LTR) patients and PWH, respectively), to determine whether freezing could influence the final outcome, as previously reported for other viruses [19,20].

**Figure 1:**
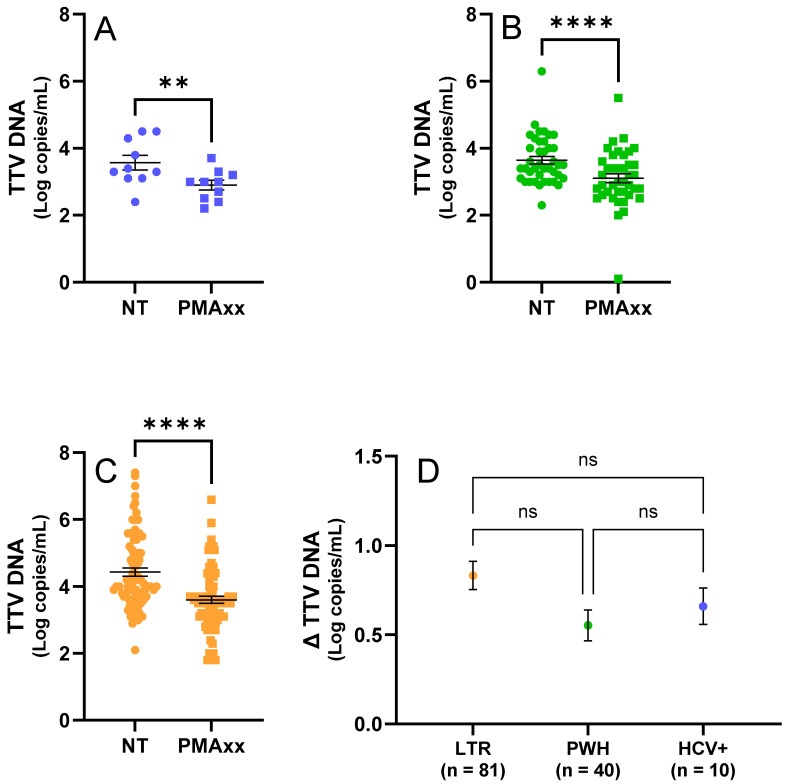
TTV results of testing samples by ddPCR before and after the PMAxx™ treatment. (**A**) Fresh samples (n. 10, in blue) untreated (NT) and treated with PMAxx™. (**B**) Frozen PWH samples (n. 40, in green), untreated (NT) and treated with PMAxx™. (**C**) Frozen LTR samples (n. 81, in orange), untreated (NT) and treated with PMAxx™. (**D**) Δ TTV DNA stratified by study population (HCV+, LTR, and PWH). Black lines indicate the mean and standard error of the mean (SEM); asterisks indicate statistical significance levels (** *p* < 0.01; **** *p* < 0.0001; ns = not significant).

In the fresh samples, a significant difference (*p* = 0.002; Figure 1A) in the TTV DNA levels was observed between the samples treated with 50 μM PMAxx™ and untreated controls, with an average reduction in the TTV DNA viremia of 0.66 Log copies/mL (SEM = ±0.10 Log copies/mL).

In the frozen samples from the LTR patients and PWH, a significant difference (*p* < 0.0001; Figure 1B,C) in the TTV DNA levels was also observed between treated and untreated samples, with an average reduction of 0.74 Log copies/mL (SEM = ±0.06 Log copies/mL). The PWH samples showed an average reduction in TTV DNA viremia of 0.55 Log copies/mL (SEM = ±0.09 Log copies/mL; Figure 1B,D), while the samples from the LTR patients showed an average reduction of 0.83 Log copies/mL (SEM = ±0.08 Log copies/mL; Figure 1C,D).

As shown in Figure 1D, no statistically significant differences in the TTV DNA levels between the treated and untreated samples (Δ TTV DNA) were found when comparing the LTR, PWH, and HCV+ groups (LTR vs. HCV+: *p* > 0.999; LTR vs. PWH: *p* = 0.083; PWH vs. HCV+: *p* = 0.659).

**Figure 2:**
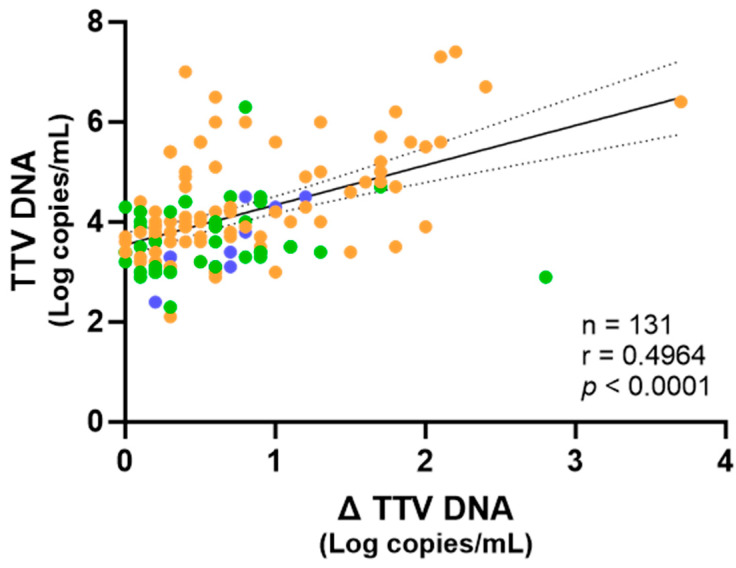
Correlation of the TTV levels in untreated samples with Δ TTV DNA between treated and untreated samples. Each point represents an individual sample. Fresh samples are depicted in blue, while frozen samples are depicted in green and orange, from PWH and LTRs, respectively. The regression line, confidence interval, *p*-value, and r value are shown.

Importantly, linear regression analysis conducted on the combined dataset including all samples revealed that untreated TTV DNA viremia was positively correlated with the Δ TTV DNA (Figure 2).

### 2.2. Correlation Between TTV DNA Levels After PMAxx™ Treatment and Immune Responses in PWH and LTR Patients

**Figure 3:**
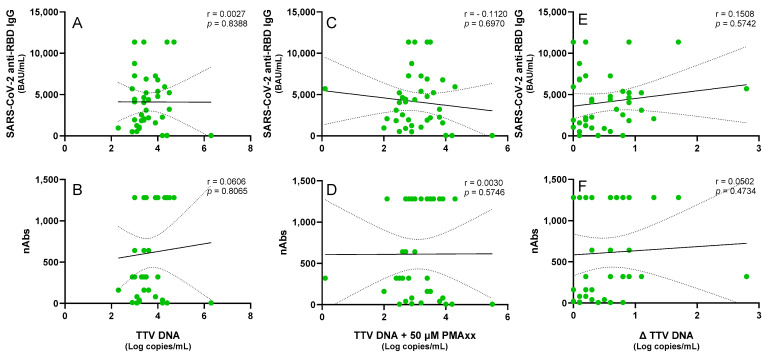
Correlations of the untreated and PMAxx™-treated TTV DNA levels with the anti-SARS-CoV-2 IgG levels and nAbs responses in the PWH. Correlations between the TTV DNA and the (**A**) SARS-CoV-2 anti-RBD IgG and (**B**) nAbs. Correlations between the PMAxx™-treated TTV DNA and the (**C**) SARS-CoV-2 anti-RBD IgG and (**D**) nAbs. Correlations between the Δ TTV DNA and the (**E**) SARS-CoV-2 anti-RBD IgG and (**F**) nAbs. Each point represents an individual sample. Regression lines, confidence intervals, *p*-values, and r values are shown.

To determine whether the PMAxx^TM^ treatment could prevent misinterpretations in the immune competence evaluations and consequently improve clinical decision-making, TTV levels before and after the PMAxx™ treatment were correlated with anti-SARS-CoV-2 IgG and nAbs responses in the PWH and LTR patients. As shown in Figure 3, no significant correlation was observed in the PWH between the pre-treated TTV DNA and levels of SARS-CoV-2 IgG (r = 0.003, *p* = 0.839) and nAbs (r = 0.061, *p* = 0.806) responses. Statistically not significant results were also observed when either the post-PMAxx™ treatment TTV DNA levels (SARS-CoV-2 anti-RBD IgG: r = −0.112, *p* = 0.697; nAbs: r = 0.003, *p* = 0.575) or Δ TTV DNA (SARS-CoV-2 anti-RBD IgG: r = 0.151, *p* = 0.574; nAbs: r = 0.050, *p* = 0.473) were used for the correlations.

**Figure 4:**
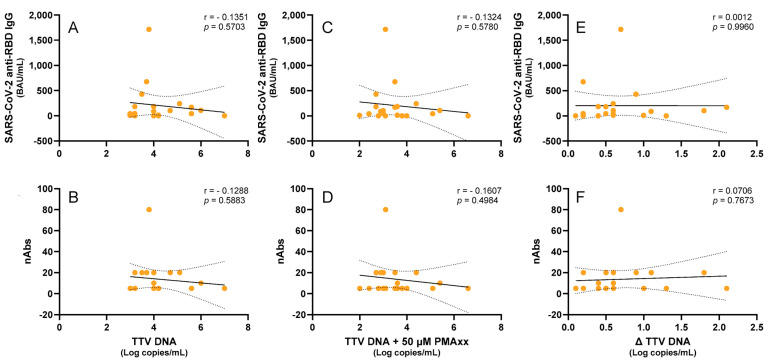
Correlations of the untreated and PMAxx™-treated TTV DNA levels with the anti-SARS-CoV-2 IgG levels and nAbs responses in the LTR patients. Correlations between the TTV DNA and the (**A**) anti-SARS-CoV-2 RBD IgG levels and (**B**) nAbs titers. Correlations between the PMAxx™-treated TTV DNA and the (**C**) anti-SARS-CoV-2 RBD IgG levels and (**D**) nAbs titers. Correlations between the Δ TTV DNA and the (**E**) SARS-CoV-2 anti-RBD IgG and (**F**) nAbs. Each point represents an individual sample. Regression lines, confidence intervals, *p*-values, and r values are shown.

Subsequently, a cohort of LTR patients was examined. Although recent studies have shown a correlation between the TTV DNA levels and immune responses in these patients [7], we selected a group of 20 patients where this correlation was absent in order to investigate whether the PMAxx™ treatment could induce any changes. Thus, a negative correlation trend, which was not statistically significant, was chosen between the TTV DNA and SARS-CoV-2 anti-RBD IgG (r = −0.135, *p* = 0.570) or nAbs (r = −0.129, *p* = 0.588). When TTV-PMAxx™ was correlated, a similar trend of negative correlation with SARS-CoV-2 anti-RBD IgG (r = −0.132, *p* = 0.578) or nAbs (r = −0.161, *p* = 0.498) was observed. These trends were further diminished when considering Δ TTV DNA (SARS-CoV-2 anti-RBD IgG: r = 0.001, *p* = 0.996; nAbs: r = 0.071, *p* = 0.767; Figure 4).

### 2.3. TTV DNA—PMAxx™ in LTRs over Time

**Figure 5:**
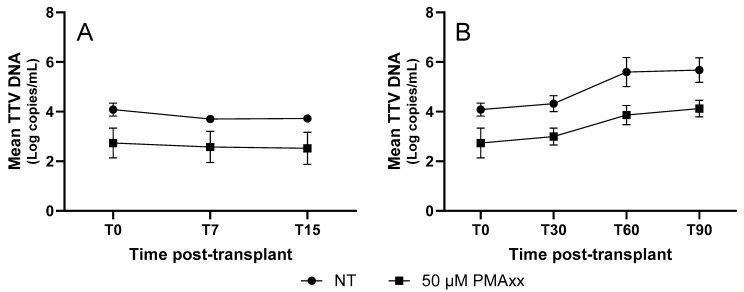
TTV DNA kinetics of untreated and PMAxx™-treated samples from 5 LTR patients. (**A**) TTV DNA levels within 15 days post-transplantation. (**B**) TTV DNA levels until 90 days post-transplantation. Each point represents the mean Log copies/mL from five patients; error bars indicate the standard error of the mean (SEM). NT, untreated samples.

To evaluate whether the time since transplantation affects the mean difference in the TTV DNA levels between the PMAxx™-treated and untreated samples, five LTRs were followed for up to 90 days post-transplantation. Samples were collected on the day of transplantation (T0), and on days 7 (T7), 15 (T15), 30 (T30), 60 (T60), and 90 (T90). As shown in Figure 5, the TTV DNA treated with PMAxx™ exhibited a temporal trend similar to that of the untreated TTV DNA but consistently showed lower DNA levels, with an average mean reduction of 0.99 Log copies/mL.

## 3. Discussion

This study investigated the use of PMAxx™, a virion viability dye known for discrimination between intact and compromised viral particles [19,20,24,25,26] in the measurement of TTV DNA in serum samples from several patient populations. Our findings indicate that a percentage of circulating TTV DNA originated from structurally compromised or non-intact viral particles treatment since treatment with 50 µM PMAxx™ resulted in a statistically significant reduction in the detectable TTV DNA levels in both the fresh and frozen serum samples. Interestingly, these results were consistent across various clinical cohorts, such as PWH, LTRs, and HCV+ individuals, demonstrating the wide applicability of the PMAxx™-based method for improving TTV quantification.

In line with earlier reports on other viruses where PMA or PMAxx™ was used to evaluate capsid integrity [19,20,27,28,29,30], the average drop in the TTV DNA after the PMAxx™ treatment ranged from 0.55 to 0.83 Log copies/mL. These findings imply that by amplifying the DNA from lysed or non-infectious virions, conventional quantification may overestimate the TTV DNA levels. In clinical settings, this overestimation may result in incorrect immune status interpretations, especially in populations where TTV is being investigated as a biomarker for immunosuppression or immune reconstitution [31]. Notably, a significant correlation was observed between the total (untreated) TTV DNA and the change in DNA levels after PMAxx™ treatment (Δ TTV DNA), implying that higher TTV DNA may include a greater proportion of non-intact particles. However, the Δ TTV DNA did not significantly differ across the clinical groups, indicating that the structural integrity of circulating TTV may be independent of the underlying condition or immune status, at least within the limits of our sample size and study design.

The possible relationship between the TTV DNA levels, both untreated and treated with PMAxx™, and the humoral immune responses triggered by SARS-CoV-2 vaccination was also investigated in this study in two immunocompromised populations: LTRs and PWH. Because previous analyses using untreated TTV DNA had not shown a significant association with vaccine-induced antibody responses, these two groups were specifically chosen [7,8]. The rationale for including PMAxx™ treatment was to determine whether selectively quantifying DNA from intact, potentially replication-competent viral particles could enhance the predictive value of the TTV load as a biomarker of immune competence.

In the case of LTR, the cohort was deliberately chosen from a subgroup in which no correlation had previously been observed between untreated TTV DNA and a vaccine response, despite existing literature suggesting that such an association can be present in this population. This approach aimed to test whether the PMAxx™ treatment could uncover a latent correlation that might be masked by total TTV DNA measurements. In the PWH, the TTV load, even when adjusted to reflect a viable virus, did not appear to be a reliable indicator of humoral vaccine responsiveness, as no statistically significant correlations were found between the TTV DNA levels, regardless of the PMAxx™ treatment, and either the anti-RBD IgG levels or nAbs titers. Both the untreated and PMAxx™-treated samples showed a tendency toward a negative correlation between the TTV DNA levels and antibody responses in the LTR patients; however, these associations did not reach statistical significance. Overall, while the use of PMAxx™ helped to improve the measure of TTV DNA quantification, the results indicate that this approach did not substantially enhance its utility in predicting SARS-CoV-2 vaccine responses.

In addition, to evaluate whether the time since transplantation affects the mean difference in TTV DNA levels between PMAxx™-treated and untreated samples, five LTR patients were longitudinally followed. The analysis showed an average reduction of 0.99 Log copies/mL, a result very similar to that observed in the LTR cohort where time since transplantation was not considered (i.e., 0.83 Log copies/mL). Moreover, the longitudinal monitoring of these five LTR patients further demonstrates that the kinetics of PMAxx™-treated TTV DNA closely mirrored those of the untreated samples (with consistently lower TTV DNA observed in treated specimens), supporting the hypothesis that TTV replication dynamics, as measured by PMAxx™-ddPCR, remain a stable indicator of host immune activity over time, and that PMAxx™ treatment can enhance the specificity of this marker without distorting its temporal behavior. However, it does not seem to offer more insights beyond those already demonstrated with the kinetics of untreated TTV DNA.

This study had several strengths, such as the investigation of both fresh and frozen samples, the inclusion of samples from various patient populations, and the use of ddPCR for high-sensitivity quantification. However, the sample size for the correlation analyses with vaccine-induced antibodies, particularly in the transplant cohort, was relatively small, potentially limiting the power to detect significant associations.

Overall, our findings suggest that incorporating the PMAxx™ treatment into TTV quantification workflows, although effective at eliminating non-intact viral particles, did not appear to enhance the established clinical utility of this virus as a surrogate biomarker of immune status [15,32,33,34]. Further studies are warranted to validate this approach in larger cohorts and to explore its prognostic value in contexts such as post-transplant immunosuppressive management and cancer immunotherapy.

## 4. Materials and Methods

### 4.1. Specimens

Fresh and frozen serum samples were used in the experiments. Fresh samples were obtained from 10 HCV+ patients, while 81 and 40 frozen specimens were from the LTRs and PWH, respectively. The study population had a mean age of 61 years (min–max: 26–79) and 78% were male [HCV+: mean age of 53 years (min–max: 30–66) and 60% male; LTR: mean age of 57 years (min–max: 26–73) and 80% male; PWH: mean age of 64 years (min–max: 43–79) and 80% male].

The patients had received two or three doses of an mRNA-based COVID-19 vaccine (either BNT162b2 or mRNA-1273) and had available serum samples collected at the time of the first or third vaccine dose.

### 4.2. PMAxx™ Treatment

The PMAxx™ treatment was performed on 100 μL of serum samples using 50 μM of PMAxx™ Dye (Biotium, San Francisco, CA, USA [35]). The PMA-Lite™ 2.0 LED Photolysis Device (Biotium, San Francisco, CA, USA [35]) was utilized to photoactivate PMAxx™ for 30 min, following the manufacturer’s instructions, as previously described [19,20].

### 4.3. TTV DNA Quantification

TTV DNA was extracted from both the PMAxx™-treated and untreated samples using the QIAamp Viral DNA Mini Kit (Qiagen, Milano, Italy [36]), following the manufacturer’s instructions. Quantification of TTV DNA was carried out with the Bio-Rad QX200 AutoDG Digital Droplet PCR system (Bio-Rad, Hercules, CA, USA [37]), as previously described [14,15]. In brief, each ddPCR reaction was prepared in a final volume of 20 μL, containing primers at a final concentration of 0.9 μM each and a probe at a final concentration of 0.25 μM, targeting the untranslated region of the TTV genome [38,39]. Reactions were assembled using the ddPCR Supermix (Bio-Rad, Hercules, CA, USA [37]). To ensure consistent quantification, DNA from each sample was analyzed in three separate wells of the same ddPCR plates, and the results were combined for the final analysis. After the PCR reaction, the droplets were read using a QX100 droplet reader, and data analysis was performed with QuantaSoft software version 1.7.4.0917 (Bio-Rad, Hercules, CA, USA [37]). A positive and a negative control sample were included in each run. Data of the TTV DNA were analyzed using the logarithm to better highlight meaningful trends and differences in the viral load dynamics.

### 4.4. SARS-CoV-2 IgG Antibody Testing

SARS-CoV-2 anti-receptor binding domain (RBD) IgG antibodies were measured in the not-treated PMAxx^TM^ samples using the Abbott Architect SARS-CoV-2 IgG II Quant Assay™ (Abbott, North Chicago, IL, USA [40]), a chemiluminescent microparticle immunoassay (CMIA). The assay was performed on serum samples collected 2–4 weeks after the administration of the second or third COVID-19 vaccine dose, following the manufacturer’s instructions. The antibody levels were converted to binding antibody units (BAU)/mL using a multiplication factor of 0.142, in accordance with the World Health Organization SARS-CoV-2 immunoglobulin standard [41,42]. The assay had a quantification range of 1.0 to 11,360 BAU/mL, with a positivity threshold set at 7.1 BAU/mL. Participants with anti-RBD IgG levels of ≥7.1 BAU/mL were categorized as vaccine responders, while those with levels below this threshold were classified as non-responders [43]. The assay used is a validated diagnostic kit that includes its own positive and negative controls.

### 4.5. SARS-CoV-2 Microneutralization Assay

The microneutralization assay (MNA) was conducted in not-treated PMAxx^TM^ samples using the SARS-CoV-2 Wuhan-D614G strain (GISAID accession ID: EPI_ISL_568579). Serum samples, initially diluted 1:10, were heat-inactivated at 56 °C for 30 min. Twofold serial dilutions were then prepared in duplicate. Equal volumes of each serum dilution and a viral suspension containing 100 TCID50 of SARS-CoV-2 were mixed and incubated for 30 min at 37 °C in a 5% CO_2_ atmosphere. Subsequently, 100 μL of each virus–serum mixture was added to confluent Vero E6 cell monolayers in 96-well tissue culture plates. After a 48 h incubation at 37 °C and 5% CO_2_, the cytopathic effect (CPE) was assessed by light microscopy. The neutralization titer (MNA90) was defined as the highest serum dilution capable of inhibiting ≥ 90% of the CPE. The serial dilutions ranged from 1:10 to 1:1280, and titers equal to or above 1:10 were considered positive [43]. A positive and a negative control sample were included in each run.

### 4.6. Statistical Analysis

The data management and analysis, including calculations of the mean, standard error of the mean (SEM), correlation analyses, simple linear regression analyses, Mann–Whitney test, Wilcoxon test, and Kruskal–Wallis tests, were conducted using GraphPad Prism version 9.3.1 (GraphPad Software, Boston, MA, USA [44]).

## Data Availability

The data that support the findings of this study are available from the corresponding author upon reasonable request.

## Abbreviations

The following abbreviations are used in this manuscript:

ddPCR	Digital droplet PCR
LTR	Liver transplant recipient
PMA	Propidium monoazide
PWH	People with HIV
TTV	Torquetenovirus
